# A novel hACE2 knock-in mouse model recapitulates pulmonary and intestinal SARS-CoV-2 infection

**DOI:** 10.3389/fmicb.2023.1175188

**Published:** 2023-06-06

**Authors:** Xiaoyang Zhou, Weiyang Sun, Yu Zhang, Hongjing Gu, Ruixuan Wang, Peng Xie, Yunkai Zhu, Minyue Qiu, Xiaoyan Ding, Hui Wang, Yuwei Gao, Jintao Li

**Affiliations:** ^1^Department of Biosafety, School of Basic Medicine, Army Medical University, Chongqing, China; ^2^Changchun Veterinary Research Institute, Chinese Academy of Agricultural Sciences, Changchun, China; ^3^State Key Laboratory of Pathogen and Biosecurity, Beijing Institute of Microbiology and Epidemiology, AMMS, Beijing, China

**Keywords:** mouse model, intestinal infection, SARS-CoV-2, immune cells, pneumonia

## Abstract

Severe acute respiratory syndrome coronavirus 2 (SARS-CoV-2) transmission is responsible for the coronavirus disease 2019 (COVID-19) pandemic. SARS-CoV-2 uses the angiotensin-converting enzyme 2 (ACE2) receptor to enter the host, and the gastrointestinal tract is a potential infection site as this receptor is expressed on it. Multiple studies have indicated that an increasing number of COVID-19 patients presented with gastrointestinal symptoms that are highly associated with disease severity. Moreover, emerging evidence has demonstrated that alterations in the gut immune microenvironment induced by intestinal SARS-CoV-2 infection can regulate respiratory symptoms. Therefore, targeting the intestines may be a candidate therapeutic strategy in patients with COVID-19; however, no mouse model can serve as an appropriate infection model for the development of fatal pneumonia while mimicking intestinal infection. In this study, a novel human ACE2 knock-in (KI) mouse model (or hACE2-KI) was systemically compared with the popular K18-hACE2 mice; it showed differences in the distribution of lung and intestinal infections and pathophysiological characteristics. These newly generated hACE2-KI mice were susceptible to intranasal infection with SARS-CoV-2, and not only developed mild to severe lung injury, but also acquired intestinal infection. Consequently, this model can be a useful tool for studying intestinal SARS-CoV-2 infection and developing effective therapeutic strategies.

## 1. Introduction

The coronavirus disease 2019 (COVID-19) has been a global health crisis for the past few years. It is well established that the causative pathogen, severe acute respiratory syndrome coronavirus 2 (SARS-CoV-2) belonging to the order *Nidovirales*, family *Coronaviridae*, and genus *Coronavirus*, uses human angiotensin-converting enzyme 2 (hACE2) as the main receptor for initiating the infection (Hoffmann et al., 2020). Most studies have focused on clinical symptoms in the respiratory tract, as viral infection can cause acute respiratory distress syndrome and respiratory failure, which are considered the main causes of death in COVID-19 patients (Huang et al., 2020; Empson et al., 2022; Ramadori, 2022; Wang and Perlman, 2022). However, ACE2 is also highly expressed in the human intestinal tract, and a increasing number of patients presented with gastrointestinal symptoms (Liang et al., 2020; Lin et al., 2020; Nobel et al., 2020; Muus et al., 2021; Blackett et al., 2022; Freedberg and Chang, 2022; Jin et al., 2022). Furthermore, direct evidence of active SARS-CoV-2 replication in the intestines has been found in clinical samples (Xiao et al., 2020; Qian et al., 2021; Zuo et al., 2021; Cuicchi et al., 2022; Freedberg and Chang, 2022).

Multiple studies have indicated that gastrointestinal symptoms may be closely associated with disease severity in COVID-19 patients. From a clinical perspective, patients with gastrointestinal symptoms have lower mortality than those without gastrointestinal symptoms (Hajifathalian et al., 2020; Nobel et al., 2020; Livanos et al., 2021). According to previous studies, bidirectional cross-talk between the intestinal immune microenvironment and respiratory tract infections occurs during influenza infection (Trompette et al., 2018; Sencio et al., 2020). Therefore, it was hypothesized that intestinal immune status after SARS-CoV-2 infection may contribute to the regulation of COVID-19 severity. Thus, a successful small animal model capable of recapitulating the respiratory and intestinal pathology observed in COVID-19 patients is expected to be useful to investigate the role of intestinal infection in modulating COVID-19.

As SARS-CoV-2 is unable to engage mouse ACE2 as a receptor for infection, mice expressing hACE2 have been developed and widely used for COVID-19 research (Bao et al., 2020; Jiang et al., 2020; Wan et al., 2020; Dong et al., 2022; Sefik et al., 2022). However, the currently available mouse models used to recapitulate the major pathological characteristics of COVID-19 patients do not mimic the intestinal infection well. Mice transduced with replication-defective adenoviruses only transiently express hACE2 in the lungs, and virtually no viral RNA is detected in the intestines (Hassan et al., 2020; Sefik et al., 2022). Some transgenic mouse models express hACE2 in the intestines driven by strong promoters (epithelial cell cytokeratin-18 [K18], lung-ciliated epithelial cell-specific HFH4/FOXJ1, and CAG); however, hACE2 expression is not physiologically distributed in these models (Jiang et al., 2020; Asaka et al., 2021; Dong et al., 2022). Notably, these transgenic mice show high brain tropism of SARS-CoV-2, and the symptoms that lead to mortality are mostly due to central nervous system dysfunction, whereas COVID-19 is primarily a respiratory disease in humans. Recently, hACE2 knock-in (KI) mouse models have received considerable attention because they have a clear genetic background, good reproductive performance, and most importantly, appropriate hACE2 tissue distribution under the control of an endogenous promotor. However, no obvious clinical symptoms or mortality are observed, and pathological changes in the lung tissues are minimal in these models. Consequently, suboptimal virus replication in these mice may lead to a failure in establishing intestinal infection (Sun et al., 2020; Sefik et al., 2022; Winkler et al., 2022).

In this study, a novel humanized hACE2 KI mouse model was established using clustered regularly interspaced palindromic repeats (CRISPR)/Cas9. This model is susceptible to intranasal infection with SARS-CoV-2, and mild to severe lung injury as well as clear evidence of intestinal infection was observed. Hence, the hACE2-KI mouse model described in this study can be of great value to find new clues to uncover COVID-19 pathogenesis and test new therapeutics for combating SARS-CoV-2.

## 2. Materials and methods

### 2.1. Ethics statement

All procedures involving infectious viruses were conducted in a Biosafety Level 3 laboratory. All experimental procedures involving mice in this study were approved by the Laboratory Animal Welfare and Ethics Committee of the Third Military Medical University (AMUWE20201373).

### 2.2. Establishment of hACE2-KI mice

To generate hACE2 KI mouse models, eggs were collected from mated female BALB/c mice and subjected to pronuclear microinjection with a complex of Cas9 mRNA, a pair of single guide RNAs (sgRNAs) sgRNA1 and sgRNA2 (Table 1), and an hACE2 KI vector plasmid at concentrations of 20, 10, and 10 ng/μL, respectively. The microinjected zygotes were subsequently implanted into pseudopregnant recipient mice. F0 founder mice were identified using two pairs of primers hACE2-F2/R2 and hACE2-F1/R1 (Table 1), which covered the 5′ and 3′ junction regions of hACE2 KI allele, respectively. Animals with positive PCR products were further confirmed by sequencing with primers R5 and R3 (Table 1) for 5′ and 3′ junction regions, respectively. The expected size of amplicons was 3,800 bp and 3,900 bp, respectively. Genotype-positive founders were backcrossed with wild-type (WT) BALB/c mice to produce generation F1, which were screened and positive individuals were self-bred. DNA samples of homozygous mice were subjected to Southern blotting to confirm correct insertion using 5′ and 3′ probes. Two endonucleases *Bam*HI (New England Biolabs, Ipswich, MA, United States; R0136) and *Mfe*I (New England Biolabs; R0589) were used to digest the DNA to identify correct insertion and no random recombinant. The expected fragment sizes for Southern blotting were 5′ probe-*Bam*HI: 4.54 kb-WT, 3.00 kb-mutation (MT) and 3′ probe-*Mfe*I: 10.83 kb-WT, 4.25 kb-MT, respectively. Primer pairs of 5′ probe (5′ probe-F/R) and 3′ probe (3′ probe-F/R) are shown in Table 1.

**Table 1:** Primers and probes used for PCR or real-time qPCR analysis.

Primer/Probe/sgRNA name	Primer/Probe/sgRNA sequence
hACE2-F2	5′-GCTTCCACTCCTTATTAGCCTAGTG-3′
hACE2-R2	5-TATCCTCACTTTGATGCTTTGGTC-3′
hACE2-F1	5′-TGAATAATGCTGGGGACAAATGG-3′
hACE2-R1	5′-GAGGATAGAATTGGTTCTTAGGAAGG-3′
5′ Probe-F	5′-TCTTACACTCTGGGAATGAGGACACG-3′
5′ Probe-R	5′-GATGCTTCCTGTGTGGCTTTGGTAA-3′
3′ Probe-F	5′-AATGTGCCTTTGGCCTCACAGTCTA-3′
3′ Probe-R	5′-GATGTCTGGCTTCTTTCTCCGTTGA-3′
sgRNA1	5′-CTTGGCATTTTCCTCGGTGAGGG-3′
sgRNA2	5′-TCTGAGCATCATCACTGTTTTGG-3′
Sequencing primer (R5)	5′-TAGTGGATACATTTGGGCAAGTG-3′
Sequencing primer (R3)	5′-AAGAGATGTCAAATCCTTAGGCAG-3′
qPCR-hACE2-F	5′-ACAGTCCACACTTGCCCAAAT-3′
qPCR-hACE2-R	5′-TGAGAGCACTGAAGACCCATT-3′
qPCR-mACE2-F	5′-TCCAGACTCCGATCATCAAGC-3′
qPCR-mACE2-R	5′-TGCTCATGGTGTTCAGAATTGT-3′
qPCR-mGAPDH-F1	5′-AGGTCGGTGTGAACGGATTTG-3′
qPCR-mGAPDH-R1	5′-GGGGTCGTTGATGGCAACA-3′
E-leader	5′-CGATCTCTTGTAGATCTGTTCTC-3′
E-reverse	5′-ATATTGCAGCAGTACGCACACA-3′
E-probe	5′-FAM-ACACTAGCCATCCTTACTGCGCTTCG-BHQ1-3′

### 2.3. Experimental animals and study design

As K18-hACE2 transgenic mice were widely used in studies of SARS-CoV-2 infection, we included this mouse model for a comparative study. All mice aged 8–10 weeks were divided into three groups: homozygous hACE2-KI transgenic mice (*n* = 19), K18-hACE2 transgenic mice (*n* = 9), and WT BALB/c mice (*n* = 19). hACE2-KI transgenic mice were prepared and bred in our laboratory and K18-hACE2 transgenic mice and WT BALB/c mice were obtained from GemPharmatech Co. Ltd. (Nanjing, China) and the Laboratory Animal Center of Army Medical University, respectively.

Mice in these groups were anesthetized and intranasally inoculated with SARS-CoV-2 delta (B.1.617.2, national number: CCPM-B-V-049-2,105-08) at a dose of 1 × 10^5^ plaque-forming units (PFU)/mouse. All mice were housed in groups, fed standard chow diets, monitored and weighed daily. Three mice in each group were euthanized at 3, 5, and 7 days post-infection (dpi) and tissues were collected for further analysis.

### 2.4. Immunochemistry assay

Collected tissues were fixed with 4% paraformaldehyde, embedded in paraffin, and sectioned at a thickness of 5 μm. Sections were dewaxed using xylene and rehydrated using a graded series of ethanol solutions. To detect hACE2 distribution in the organs of the hACE2-KI transgenic mouse model, an anti-hACE2 monoclonal antibody (Abcam, Cambridge, MA, United States; ab108209) was used as the primary antibody, and the sections were incubated with secondary antibody (Absin Bioscience, Shanghai, China; abs957), followed by visualization with a DAB Detection Kit (ZSGB Biotech, Beijing, China; ZLI-9018).

### 2.5. RNA extraction and real-time quantitative PCR

Total RNA was extracted from tissue homogenates of organs using RNAiso Plus (Takara Bio, Shiga, Japan; 9,108), and reverse transcription was performed to produce cDNA using the PrimeScript RT Reagent Kit (Takara Bio; RR047A) according to the manufacturer’s recommendations. Real-time quantitative PCR (qPCR) was performed using TB Green Premix Ex Taq II (Takara Bio; RR820A) with the LightCycler 96 System (Roche Diagnostics, Indianapolis, IN, United States). Each reaction was performed in triplicate. Relative expression levels of hACE2 were determined using PCR pairs of primers qPCR-hACE2-F, qPCR-hACE2-R and qPCR-mACE2-F, qPCR-mACE2-R (Table 1). Amplicons were normalized to *GAPDH* expression, and primer pairs qPCR-mGAPDH-F1/R1 used for *GAPDH* are shown in Table 1.

### 2.6. Protein extraction and expression analysis

Frozen tissues (30 mg) were homogenized in RIPA Lysis Buffer (Beyotime Biotechnology, Shanghai, China; P0013B) supplemented with protease and phosphatase inhibitors (Beyotime Biotechnology; P1050). The homogenates were kept on ice for 30 min and then spun at 13,000 × *g* for 15 min at 4°C. The supernatant was collected and protein concentrations were determined using a BCA kit (Beyotime Biotechnology; P0010S). Protein expression and total protein were measured with the automated capillary-based Jess system (ProteinSimple, San Jose, CA, United States). Briefly, each sample was mixed with a master mix (1× fluorescent standard, 1× sample buffer, and 40 mM dithiothreitol) and heated at 95°C for 5 min to denature the samples. Subsequently, 3 μL of the denatured proteins and 10 μL each of protein normalization solution, chemiluminescent substrate, primary antibodies, and horseradish peroxidase-conjugated anti-rabbit secondary antibodies were pipetted into the appropriate wells of the assay plate. A biotinylated ladder cartridge (12–230 kDa) was integrated into each assay. Subsequently, the plate and the capillaries were moved to the Jess machine for automated protein electrophoresis, blocking, antibody incubation, and signal detection. Chemiluminescent reactions were analyzed by Compass for SW software ver. 6.1.0 (ProteinSimple). Relative protein amounts were assessed using the corrected area of chemiluminescent peaks. The primary antibodies were human ACE2 antibody (Sino Biological, Beijing, China; 10,108-RP01) and mouse ACE2 antibody (R&D Systems, Minneapolis, MN, United States; MAB34372), and both were used at 1:50 dilution.

### 2.7. Measurements of viral loads

Viral RNA quantification was performed using real-time qPCR targeting the SARS-CoV-2 subgenomic RNA transcript for the envelope (E) gene, as previously reported (Wölfel et al., 2020). Briefly, RNA was isolated from the tissue homogenates using Viral RNA Extraction Kit (Takara Bio, 9,766). Real-time qPCR was performed using the One Step PrimeScript III RT-PCR Kit (Takara Bio, RR600A) with the primer pairs E-Leader, E-reverse, and E-probe (Table 1). The cycling conditions were one cycle at 52°C for 5 min, then 95°C for 10 s, followed by 45 cycles at 95°C for 10 s and 60°C for 30 s. pUC19-2019-nCoV-E plasmid was synthesized by Sangon Biotech Co., Ltd. (Shanghai, China) as an E gene DNA standard and the inserted base sequence reported in a previous study was used (Wölfel et al., 2020). An E gene DNA sample was also run at the same time for conversion of cycle threshold value to genomic copies using the standard curve-based method.

### 2.8. Histopathological analysis

The processed and sectioned tissues were stained with hematoxylin and eosin (H&E) according to the standard procedures of the H&E Staining Kit (Solarbio Science & Technology, Beijing, China; G1120). The histopathological changes in the tissues were examined by M8 Microscope and Scanner (PreciPoint, Freising, Germany) and analyzed by two experienced pathologists who were blinded to the samples.

### 2.9. Immunofluorescence assay

The processed sections were incubated with 3% H_2_O_2_ for 20 min to quench endogenous peroxidase. Antigen repair was performed in boiled citrate buffer (pH 6) for 10 min and non-specific staining was blocked using normal goat serum for 2 h at 37°C. The sections were then incubated with primary antibodies at 4°C overnight and subsequently incubated with secondary antibodies for 2 h. Finally, the sections were stained with 4′,6-diamidino-2-phenylindole (DAPI) (Sigma-Aldrich, St. Louis, MO, USA; 32,670) for 7 min. The primary antibodies used in this study included SARS-CoV-2 spike (S; Sino Biological Japan, Kanagawa, Japan; 40,592-R004), Ly6G (Cell Signaling Technology, Danvers, MA, USA; 87,048), CD68 (Abcam, ab125212), CD3 (Cell Signaling Technology, 78,588), and CD19 (Cell Signaling Technology, 90,176). Secondary antibodies included Alexa Fluor 594 goat anti-rabbit IgG (Thermo Fisher Scientific, Waltham, MA, United States; A11037) and Alexa Fluor 488 goat anti-rabbit IgG (Thermo Fisher Scientific, A11008). Representative sections were scanned using a digital slide scanner (PANNORAMIC MIDI, 3DHISTECH, Budapest, Hungary) with CaseViewer software (3DHISTECH).

### 2.10. Statistical analysis

All data analyzes were performed using the R package (version 4.2.1; R Foundation for Statistical Computing, Vienna, Austria). Data are presented as mean ± standard error of the mean. The log-rank test was used for survival analysis, and the statistical significance of immunofluorescence staining analysis among different mice groups was assessed by unpaired Student’s *t*-tests or Wilcoxon rank tests. Before statistical significance was assessed, the Shapiro–Wilk normality test was used to determine whether the data had a normal distribution. *p* < 0.05 was specified as the threshold of significance.

## 3. Results

### 3.1. Establishment of a humanized hACE2 KI mouse model using CRISPR/Cas9

**Figure 1:**
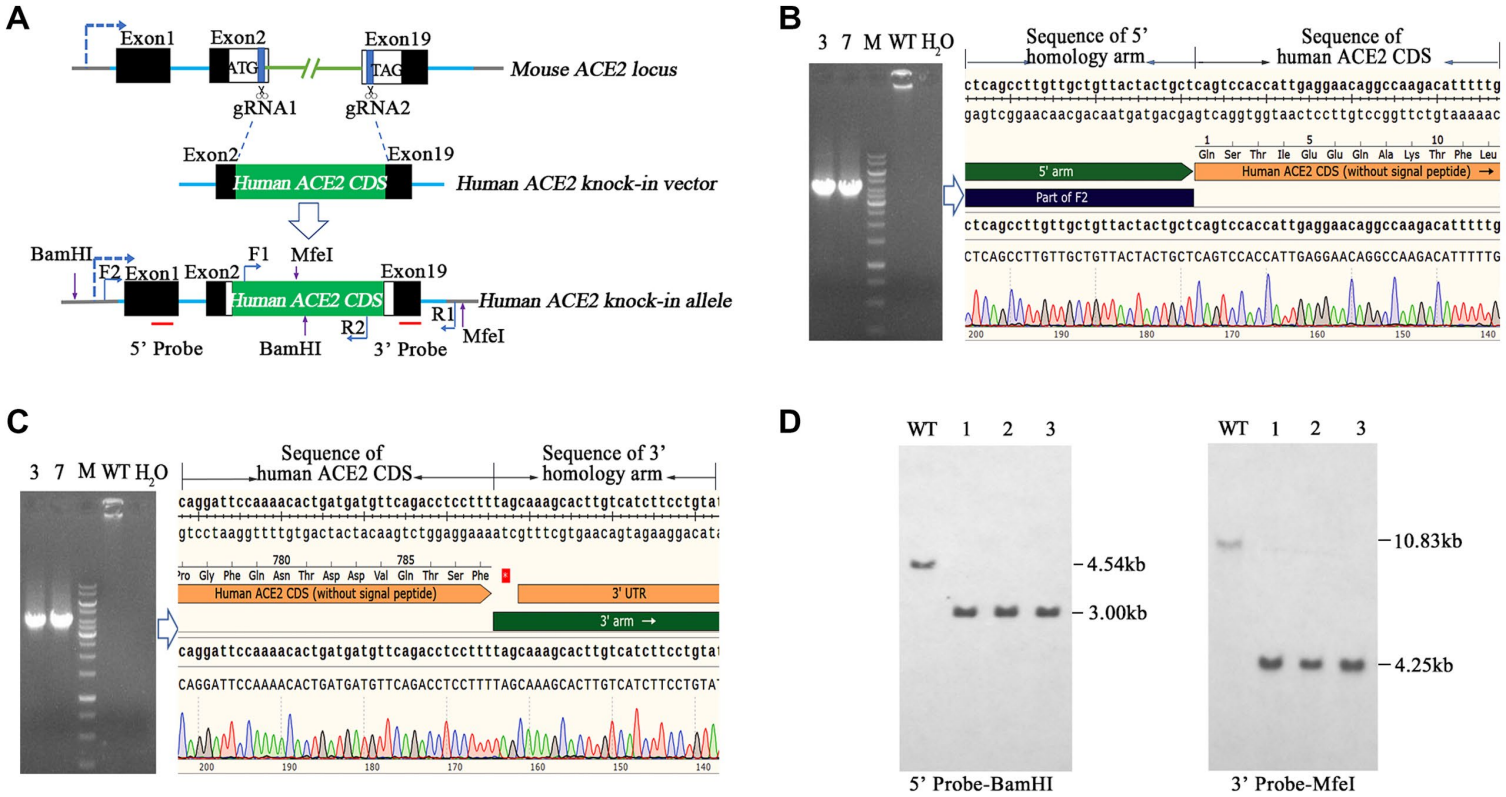
Generation and genetic characterization of human angiotensin-converting enzyme 2 (hACE2) knock-in (KI) mice. **(A)** The strategy of generation of hACE2 KI mice. A pair of single guide RNAs (sgRNAs) cutting exons 2 and 19 of mouse ACE2, respectively, was used to replace the translated exons of mouse ACE2 with the human ACE2 coding sequence (CDS) without the sequence coding signal peptide, and the human ACE2 CDS with signal peptide was placed downstream the sequence coding mouse ACE2 signal peptide as a result. Black boxes: untranslated exonic sequences of mouse ACE2; white boxes: translated exonic sequences of mouse ACE2; green box: human ACE2 CDS without signal peptide; blue lines: introns of mouse ACE2; the arrow in dotted line: the transcription direction; F1, F2, R1, and R2 show the positions of primers used for genetic screening of hACE2 KI mice; red lines indicate the locations of probes for Southern blotting. **(B,C)** Transgenic founder mice were subjected to genetic screening by PCR using primer pairs F2/R2 (B) and F1/R1 **(C)**, which covered the 5′ and 3′ junction regions of hACE2 KI allele, respectively. The PCR products were sequenced and the sequence chromatography images are shown. **(D)** Southern blotting of homozygous hACE2 KI individuals. The genomic DNAs of the homozygous individuals were completely digested by the endonucleases *Bam*HI and *Mfe*I, and then subjected to Southern blotting using the 5′ and 3′ probes shown in A, respectively. The sizes of Southern blot bands with the 5′ probe were 4.54 kb in the wild-type (WT) allele and 3.00 kb in the hACE2 KI allele, and those with 3′ probe were 10.83 kb in the WT allele and 4.25 kb in the hACE2 KI allele. WT: wild-type mouse genomic DNA; ~1–3: homozygous KI individuals.

Here, we aimed to establish a human ACE2 mouse model using CRISPR/Cas9 KI technology. As shown in Figure 1A, the mouse endogenous ACE2 coding sequence (CDS) was replaced with the human ACE2 coding sequence (CDS) without the sequence coding signal peptide via CRISPR/Cas9-mediated DNA homologous recombination by double cutting with a pair of sgRNAs, namely sgRNA1 and sgRNA2. The pair of sgRNAs precisely cut 3′ downstream of the start codon (ATG) and 5′ upstream of the termination stop codon (TAG) of mouse endogenous *ACE2*, and the hACE2 CDS was used to precisely replace the mouse endogenous *ACE2* CDS *in situ* and was placed under the drive of the mouse endogenous *ACE2* promoter. Human ACE2 CDS (without the signal peptide) was placed precisely between the start codon and stop codon of the endogenous mouse ACE2 gene, so that the inserted human ACE2 gene could be physiologically distributed in these humanized mice. As described in the Materials and Methods section, transgenic founder mice were subjected to genetic screening by PCR using primer pairs of hACE2-F2/R2 (Figure 1B) and hACE2-F1/R1 (Figure 1C), which covered the 5′ and 3′ junction regions of hACE2 KI allele, respectively. Animals with positive PCR products were further confirmed by sequencing and the sequence chromatography images of the 5′ and 3′ junction regions are shown in Figures 1B,C, respectively. The mice carrying the correct gene were backcrossed with WT mice, the F1 offspring were screened, and positive individuals were self-bred. Homozygous hACE2 KI individuals were subjected to Southern blotting to confirm correct insertion and off-targeting. The genomic DNA of the homozygous individuals was completely digested by the endonucleases *Bam*HI and *Mfe*I, and then subjected to Southern blotting using the 5′ and 3′ probes shown in Figure 1A, respectively. The sizes of Southern blot bands with 5′ probe were 4.54 kb in the WT allele and 3.00 kb in the hACE2 KI allele, and those with 3′ probe were 10.83 kb in the WT allele and 4.25 kb in the hACE2 KI allele (Figure 1D). As expected, none of the three homozygous mice showed random insertion and off-targeting (Figure 1D).

**Figure 2:**
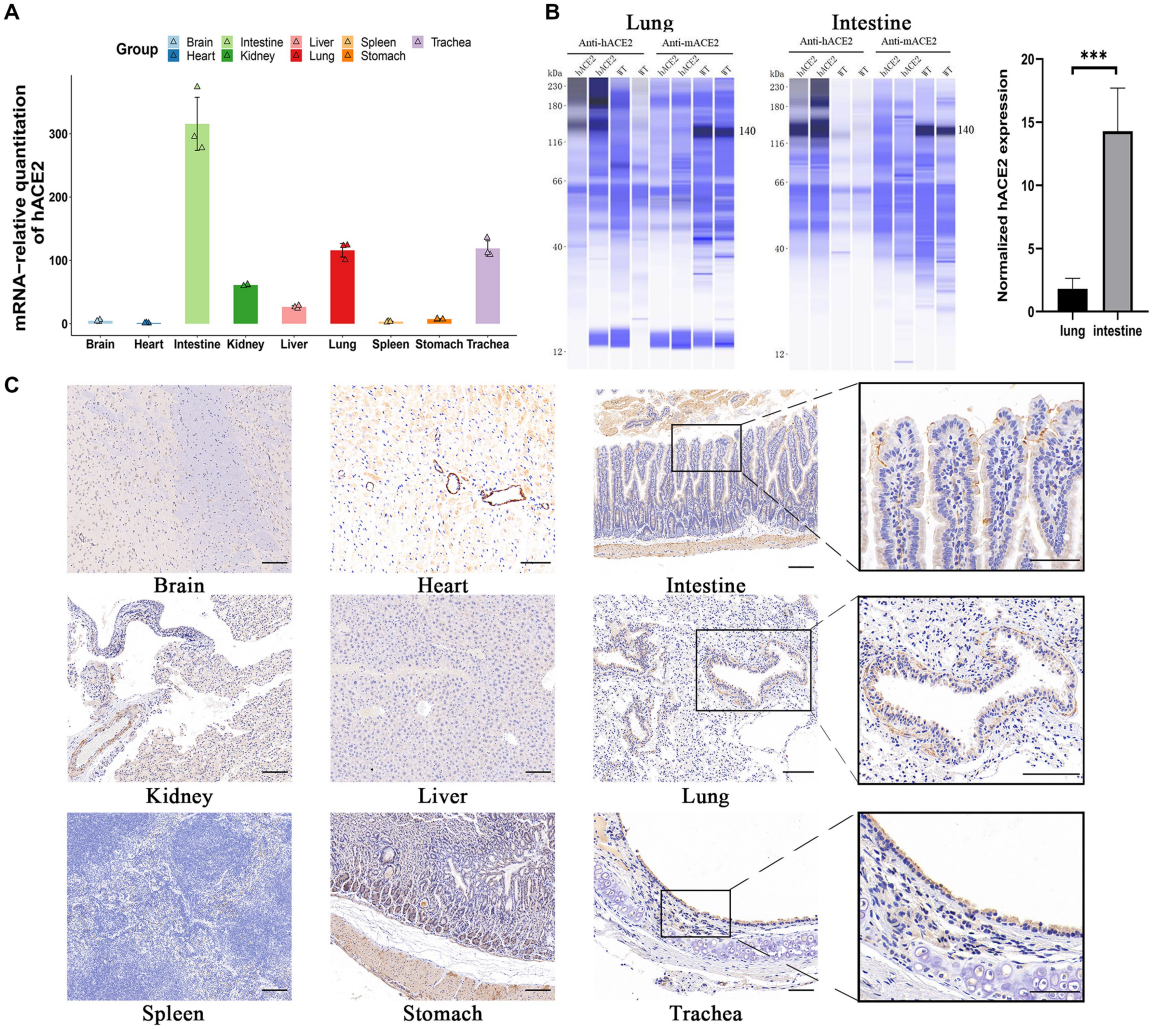
Expression of hACE2 in hACE2-KI mouse model. **(A)** The expression pattern of hACE2 mRNA in different organs including brain, heart, intestines, kidneys, liver, lungs, spleen, stomach, and trachea was detected by real-time quantitative PCR (qPCR) assay. **(B)** Representative immunoblotting image shows the expression of the ACE2 protein in mouse intestine and lung tissues normalized to total protein. Human ACE2 was highly expressed in the lungs and intestines of hACE2-KI mice, while mouse ACE2 protein was detected in WT mice but not in hACE2-KI mice. **(C)** Immunochemistry results of the hACE2 expression in different organs. The intestines, lungs, and trachea sections are shown in high-power magnification; scale bar: 100 μm. Statistical analysis was performed using unpaired Student’s *t*-test or Wilcoxon rank test. NS: not significant; *p < 0.05; **p < 0.01; ***p < 0.001.

To investigate hACE2 expression patterns, real-time qPCR was used to measure the mRNA levels in different organs. As shown in Figure 2A, hACE2 was mainly expressed in the intestines, lungs, and trachea. The expression of human ACE2 protein in mouse intestine and lung tissues was detected using the capillary-based Jess system (ProteinSimple) and Total Protein Normalization analysis. Immunoblotting of the proteins showed that hACE2 was highly expressed in the lungs and intestines of hACE2-KI mice, while mouse ACE2 protein was detected in WT mice but not in hACE2-KI mice. Furthermore, the expression level of human ACE2 in the intestinal tracts of hACE2-KI mice was about 15-fold higher than that in the lungs (Figure 2B). Immunohistochemical analysis revealed robust hACE2 expression in the intestinal brush border, lung bronchi, and tracheal epithelial cells (Figure 2C). Thus, a mouse model that highly expresses hACE2 in the intestines, lungs, and trachea was established successfully.

### 3.2. Intranasal inoculation of SARS-CoV-2 induces pulmonary and intestinal infection

**Figure 3:**
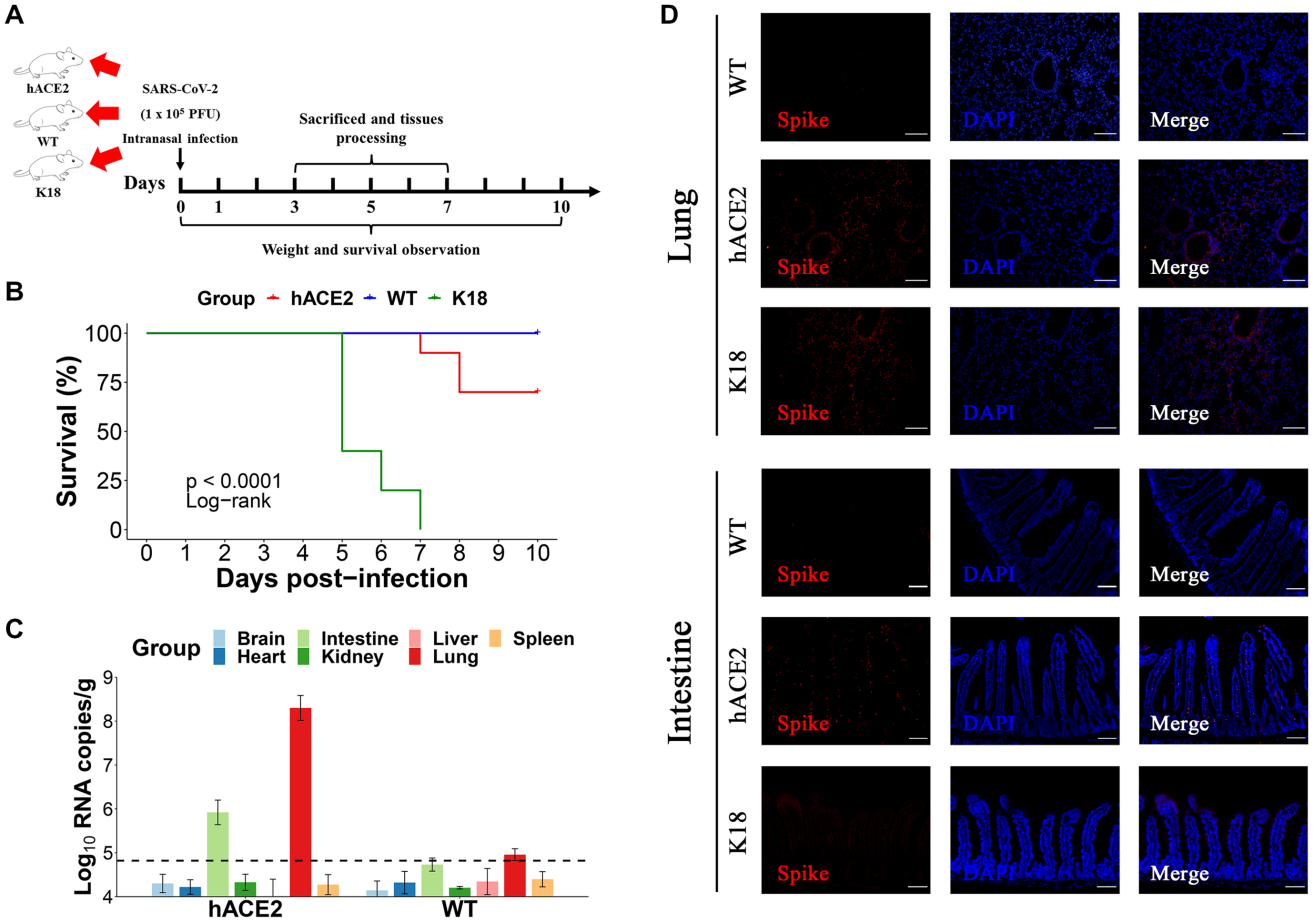
Intranasal infection of severe acute respiratory syndrome coronavirus 2 (SARS-CoV-2) in hACE2-KI mice. **(A)** The hACE2-KI, WT, and K18-hACE2 mice received intranasal SARS-CoV-2 infection at a dose of 1 × 10^5^ plaque-forming units (PFU)/50 μL per mouse, and three mice from each group were euthanized at 3, 5, and 7 days post-infection (dpi) for tissue collection. **(B)** Survival of hACE2-KI (red), WT (blue), and K18-hACE2 (green) mice were observed for 10 days. The statistical difference was evaluated by log-rank test. **(C)** Viral mRNA levels in brain, heart, kidney, lung, intestines, liver, and spleen are shown. The dotted lines indicate the detection limit. **(D)** Immunofluorescence staining analysis for the spike protein (S) (red) of SARS-CoV-2 in lungs and intestines; scale bar: 100 μm.

To further estimate the susceptibility of hACE2-KI mice to SARS-CoV-2, groups of hACE2-KI mice (*n* = 19) and K18-hACE2 mice (*n* = 9) were intranasally challenged with 1 × 10^5^ PFU of SARS-CoV-2. WT BALB/c mice (*n* = 19) that received the same viral dose were used as negative controls. All mice were monitored daily for survival or euthanized at various dpi for tissue collection to measure viral copy numbers and observe histopathological changes (Figure 3A). It was found that 30% (3/10) of hACE2-KI mice died at 7–10 dpi and 100% of K18-hACE2 transgenic mice died at 5–7 dpi (6/6), whereas all WT mice survived until the end of follow-up at 10 dpi (Figure 3B). These results were consistent with previous findings that state that the K18-hACE2 mouse is a lethal model. In contrast to K18-hACE2 mice, our established hACE2-KI model mice showed lower mortality. Next, we measured the viral infection pattern. As shown in Figure 3C, viral RNA replication was observed in the lungs and intestines of hACE2-KI mice, and RNA copies were below the detection limit in the brain, heart, kidneys, liver, and spleen. As expected, the S protein was detected with immunofluorescence staining in the lung sections of both hACE2-KI and KI-18 mice. Furthermore, only the hACE2-KI mice showed S protein-positive cells in the intestinal sections (Figure 3D). These results indicate that the intranasal inoculation of SARS-CoV-2 could induce pulmonary and intestinal infection in our mouse model.

### 3.3. Histopathological changes in lungs and intestines after SARS-CoV-2 infection

**Figure 4:**
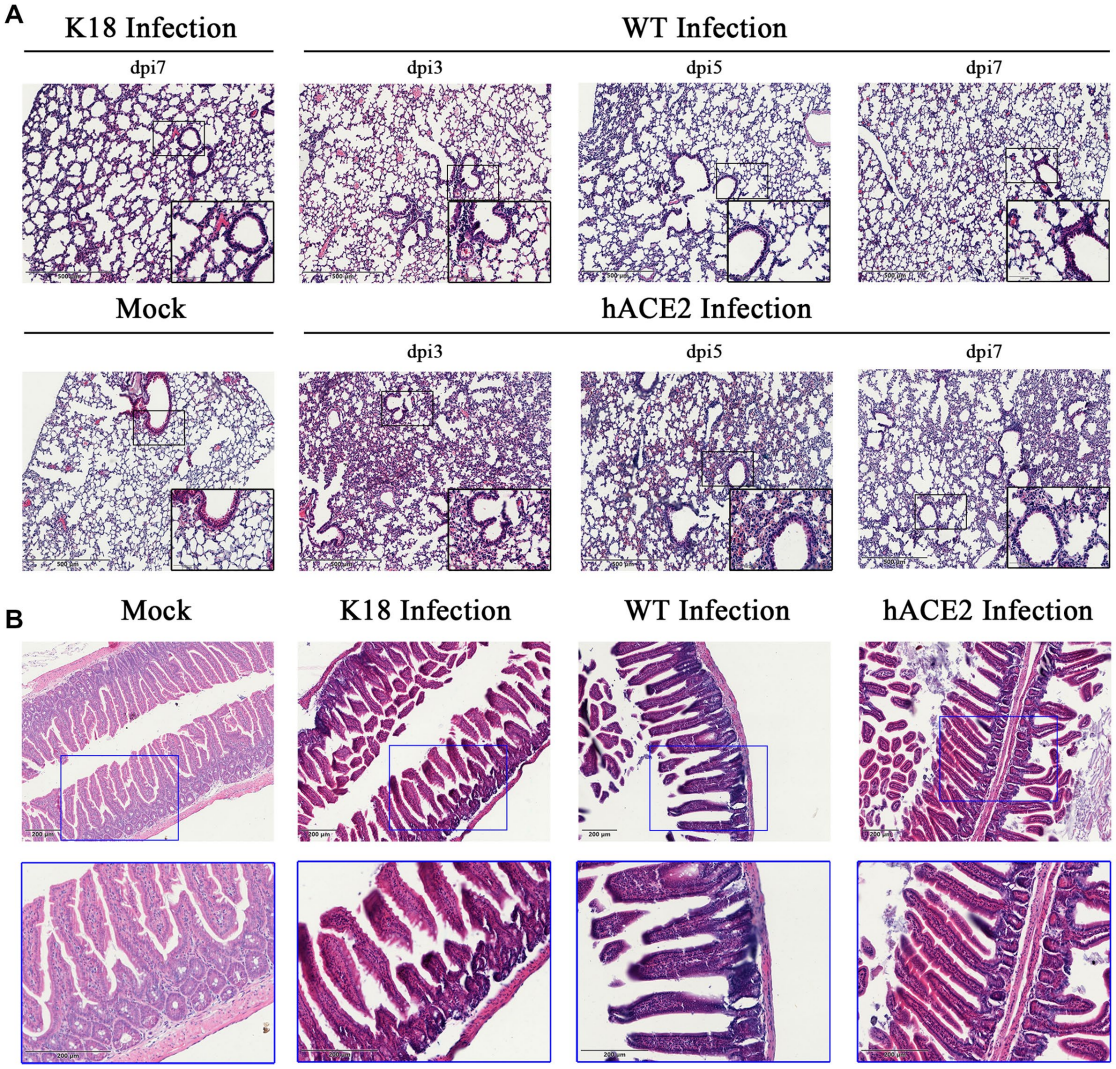
Histopathological changes in hACE2-KI mice infected with SARS-CoV-2. **(A)** Hematoxylin and eosin (H&E) staining of lung sections from hACE2-KI and WT mice after intranasal inoculation with 1 × 10^5^ PFU per mouse at 3, 5, and 7 dpi. K18-hACE2 lung tissue collected at 7 dpi is shown. **(B)** H&E staining of intestine sections from hACE2-KI, WT, and K18-hACE2 mice after intranasal inoculation with 1× 10^5^ PFU per mouse at 7 dpi. Images are representative of each study group.

Regarding the histopathological changes in lung tissues, H&E staining results showed that hACE2-KI mice acquired different levels of pneumonia in a time-dependent manner, characterized by a large amount of inflammatory cell infiltration, alveolar wall, and alveolar septa thickening (Figure 4A). As shown in the pathological sections, hACE2-KI mice displayed the most severe pneumonia at 3 dpi. In addition to inflammatory cell infiltration in the alveolar septa, alveolar congestion, peripheral parenchymal collapse, and ruptured septa were also observed. At 5 dpi, the damage progressed into more diffuse lesions with extensive infiltration of inflammatory cells into the alveolar cavities. This implies that the immune response remained high at this stage. At 7 dpi, mice that recovered from the infection were characterized by descending inflammatory cells in the alveolar septa and interstitial locations. Notably, the histopathological changes in hACE2-KI mice were comparable with those in K18-hACE2 transgenic mice during the same infection course. According to the histopathological observations in lung tissues, we confirmed that our infected mouse model developed typical pneumonia characteristics comparable to the K18-hACE2 mice. Regarding intestinal histopathological changes, mild inflammatory cell infiltration was identified in the intestinal lamina propria. Additionally, thinning of the muscular layer and intestinal epithelial injury were observed in our established model (Figure 4B). These findings imply that the hACE2-KI mice acquired intestinal injury after SARS-CoV-2 infection, which was consistent with the detected viral load in the intestine.

### 3.4. Immune responses in the lungs and intestines of hACE2-KI mice

**Figure 5:**
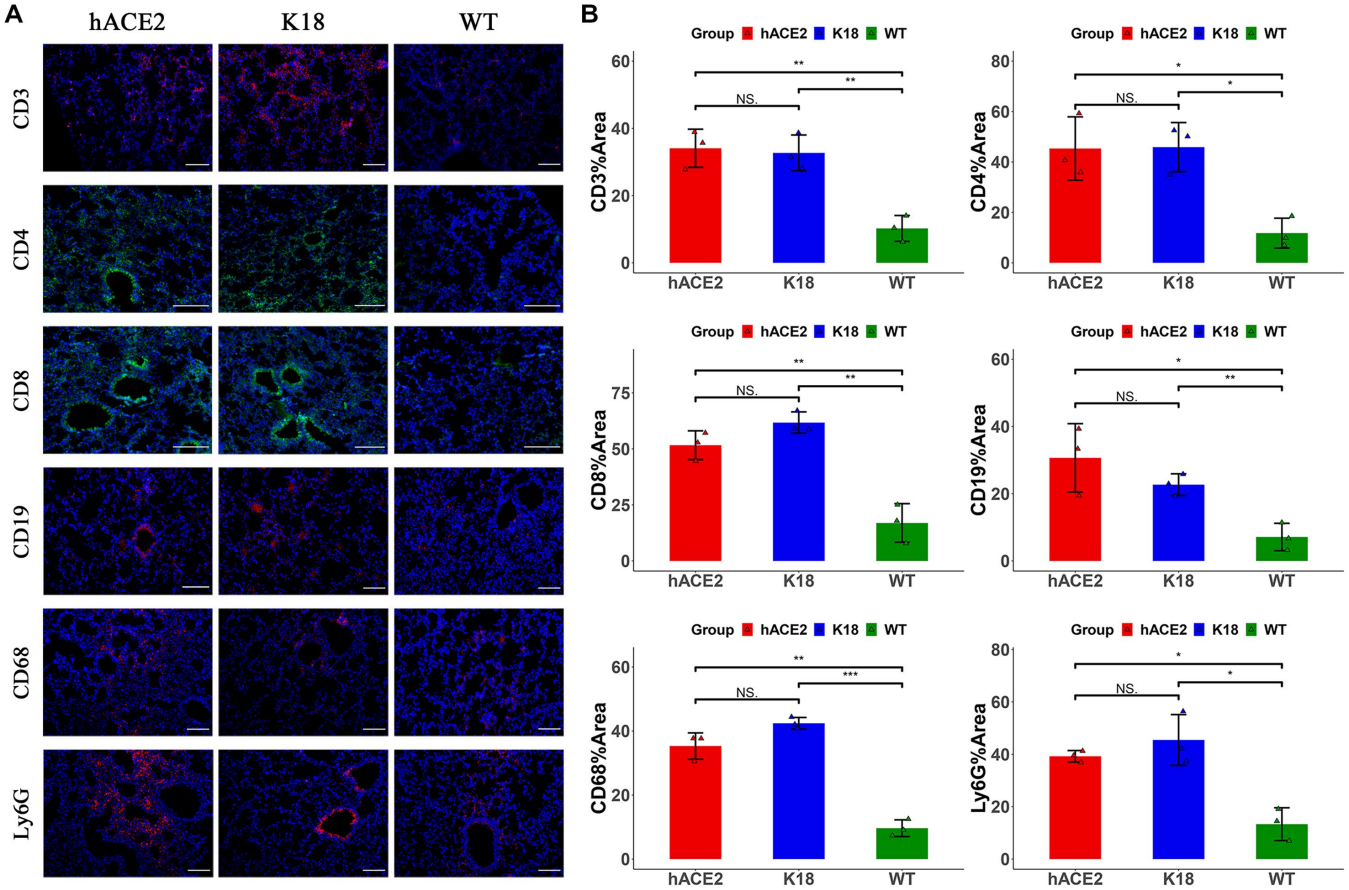
Characterization of immune responses in lungs of hACE2-KI mice infected with SARS-CoV-2. **(A)** Representative images of immunofluorescence staining analysis for the immune cells in lung tissues collected at 7 dpi. Cell markers: CD3, CD19, CD68, and Ly6G (red); CD4, CD8 (green). Scale bar: 100 μm. **(B)** Semi-quantitative analysis of area fraction of immune cells with immunofluorescence staining (*n* = 3 fields per group). Statistical analysis was performed using unpaired Student’s *t*-test or Wilcoxon rank test. NS: not significant; *p < 0.05; **p < 0.01; ***p < 0.001.

To investigate the infiltration of specific inflammatory cells, immunofluorescence assays were performed to identify CD3^+^ T lymphocytes, CD4^+^ T lymphocytes, CD8^+^ T lymphocytes, CD19^+^ B lymphocytes, CD68^+^ macrophages, and Ly6G^+^ neutrophils. Among inflammatory cells, T lymphocytes were diffusely distributed in the lungs, whereas neutrophils were mainly found in the peri-bronchus (Figure 5A). These results demonstrate that both hACE2-KI and K18-hACE2 mice exhibited elevated immune responses in the lung tissues compared with the WT group (Figure 5B). Interestingly, there were no remarkable differences in inflammatory cell counts between hACE2-KI and K18-hACE2 mice, indicating that SARS-CoV-2 infection in hACE2-KI mice induced pneumonia, similar to that described in K18-hACE2 mice.

**Figure 6:**
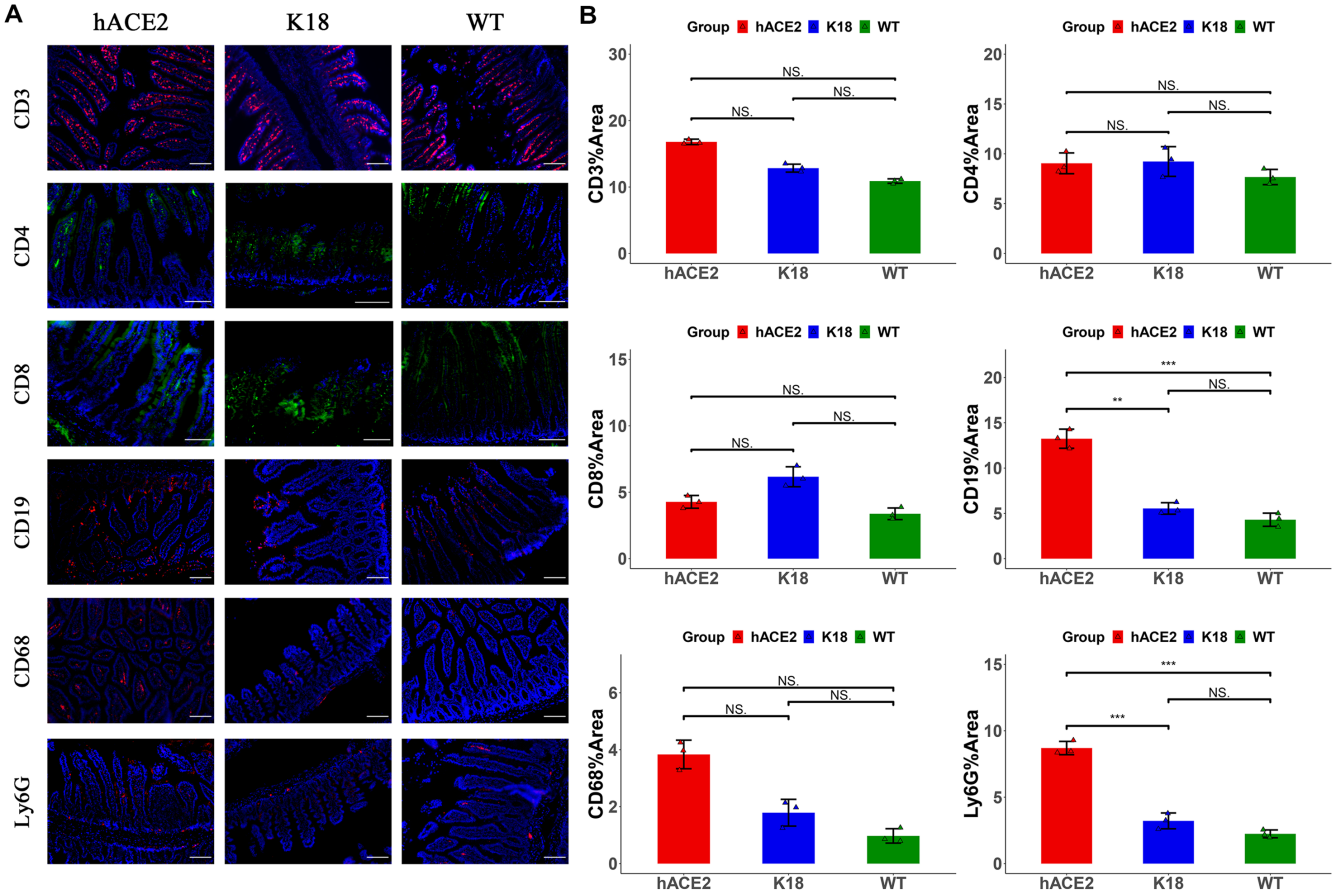
Characterization of immune responses in intestines of hACE2-KI mice infected with SARS-CoV-2. **(A)** Representative images of immunofluorescence staining analysis for the immune cells in intestinal tissues collected at 7 dpi. Cell markers: CD3, CD19, CD68, and Ly6G (red); CD4, CD8 (green). Scale bar: 100 μm. **(B)** Semi-quantitative analysis of area fraction of immune cells with immunofluorescence staining (*n* = 3 fields per group). Statistical analysis was performed using unpaired Student’s *t*-test or Wilcoxon rank test. NS: not significant; *p < 0.05; **p < 0.01; ***p < 0.001.

In the intestines, the main infiltrated immune cells were T lymphocytes (Figure 6A); however, there were no notable differences in CD3^+^, CD4^+^ and CD8^+^ T lymphocyte counts among the hACE2-KI, K18-hACE2, and WT mice groups (Figure 6B). When comparing infiltrated immune cell counts, only hACE2-KI mice exhibited elevated CD19^+^ B lymphocytes and Ly6G^+^ neutrophils (Figure 6B). Therefore, this study provided evidence of reduced inflammatory responses in the intestinal tract, which is in contrast to the significant inflammatory responses found in lung tissues. We have summarized the main similarity and differences between hACE2-KI and K18-hACE2 mouse models found in this study (Table 2).

**Table 2:** Comparisons of similarity and difference between hACE2-KI and K18-hACE2 mouse model.

Characteristics		
	hACE2-KI model	K18-hACE2 model
Promotor of hACE2 expression	Mouse endogenous promoter	Human keratin 18 promoter
Expression of hACE2 in tissues	Intestine, lung, trachea and kidney	Brain, trachea, lung, and kidney (Dong et al., 2022)
Mortality after SARS-CoV-2 infection	30% within 10 dpi at 10^5^ PFU	60–100% within 10 dpi at 2 × 10^1^–10^5^ PFU (Dong et al., 2022)
Viral infection pattern in tissues	Lung and intestine	Brain, trachea and lung (Dong et al., 2022)
Immune responses in lung	Both exhibited elevated immune responses in the lung tissues, but there were no remarkable differences in inflammatory cell counts	
Immune responses in intestine	Higher CD19+ B lymphocytes and Ly6G+ neutrophils in hACE2-KI model	

## 4. Discussion

Although most people have received vaccinations for COVID-19, SARS-CoV-2 has been spreading at an alarming rate. Therefore, the development of small animal models that could show COVID-19 clinical manifestations similar to those in humans is essential for enhancing the understanding of candidate therapeutics. In the present study, we report a novel mouse model in which the CDS of endogenous ACE2 was precisely replaced with that of human ACE2 *in situ* using CRISPR/Cas9 technology. Driven by the endogenous mouse ACE2 promoter, the expression of hACE2 was assumed to be physiologically distributed in crosses tissues. Immunochemistry, real-time qPCR, and immunoblotting were performed to confirm this hypothesis, and it was found that hACE2-KI was highly expressed in the lungs, trachea, and intestines. Interestingly, analyzes of single-cell transcriptomic data in humans also revealed that ACE2 and TMPRSS2 were co-expressed in the intestines (Zhang et al., 2020). High hACE2 expression that is commonly observed in the brains of K18-hACE2 mice was not detected in this established model (Gan et al., 2021; Dong et al., 2022). In contrast, the robust hACE2 expression identified in the intestinal brush border was consistent with the finding that human intestinal tissues also show a high hACE2 expression (Hamming et al., 2004). It is worth mentioning that this tissue distribution pattern was not observed in other mouse models used for the SARS-CoV-2 research (Bao et al., 2020; Jiang et al., 2020; Asaka et al., 2021; Sefik et al., 2021, 2022; Winkler et al., 2022).

Furthermore, the susceptibility of this model to SARS-CoV-2 was evaluated at a dose of 1 × 10^5^ PFU/mouse. Following infection, the mice exhibited a considerable reduction in body weight, which could serve as a good indicator for monitoring disease progression. In survival analysis, this mouse model showed a certain ratio of fatal cases. In comparison, no obvious clinical symptoms or mortality was observed in other hACE2 transgenic models that did not seem to recapitulate severe diseases. Although the K18-hACE2 model had a higher mortality rate, most of these mice showed neuroinvasion and high titer of viruses (demonstrating viral replication) in the brain, which was assumed to be the primary cause of lethality (Jiang et al., 2020; Rathnasinghe et al., 2020; Zhang et al., 2021; Dong et al., 2022). This is in contrast with the findings that most COVID-19 patients died due to lung injury (Hu et al., 2021). Notably, evidence of enteric infection, which is seldom identified in other mouse models, was also found and this may be due to the physiological distribution of hACE2 expression in the intestines.

To further investigate the pathological damage, H&E staining of lung and intestinal tissues was performed. After SARS-CoV-2 infection, hACE2-expressing mice showed signs of pneumonia accompanied by immune cell infiltration. Immunofluorescence assays demonstrated that the extent of the inflammatory response in this model was comparable with that observed in K18-hACE2 mice. These results indicate that this mouse model successfully developed lung damage, which is a common histopathological change that manifests in COVID-19 patients (Bradley et al., 2020; D’Onofrio et al., 2022). In addition, damage in the intestines with limited immune cell infiltration was demonstrated. Compared with K18-hACE2 and WT mice, this model only exhibited a slightly higher proportion of CD19^+^ B lymphocytes and Ly6G^+^ neutrophils. Another study, which included clinical cohorts, also observed evidence of reduced inflammatory responses in the intestinal tract (Livanos et al., 2021). The attenuation of inflammation is consistent with data from autopsy studies conducted on COVID-19 patients (Skok et al., 2021).

Notably, recent clinical studies have reported that an increasing proportion of COVID-19 patients develop gastrointestinal symptoms (Chen et al., 2020; Jin et al., 2020; Redd et al., 2020; Blackett et al., 2022; Freedberg and Chang, 2022; Jin et al., 2022) and direct evidence of active SARS-CoV-2 replication has been found in the intestines (Qian et al., 2021; Zuo et al., 2021; Cuicchi et al., 2022). Intestinal infection can induce the infiltration of plasma cells and lymphocytes in the intestinal mucosa of COVID-19 patients (Xiao et al., 2020). Consistent with these reports, elevated levels of CD19^+^ B lymphocytes and infiltration of CD3^+^ T lymphocytes were observed. In addition, intestinal immune responses induced by enteric infections may regulate lung symptoms through the gut-lung axis (Yang et al., 2021). It is reasonable to assume that targeting the intestines may be a candidate therapeutic strategy for patients with COVID-19. However, there is no appropriate mouse model that can mimic the intestinal infection process with accurate physiological manifestations, and the current *in vitro* models used for SARS-CoV-2 intestinal infection are human cell lines and organoids (Stanifer et al., 2020; Zhou et al., 2020). Consequently, it would be of great benefit to develop a COVID-19 mouse model that can be used to study intestinal infection.

Emerging evidence has demonstrated that the intestinal immune microenvironment can be regulated by many factors excluding gut immune cells, such as microbiome composition and microbial metabolites (Haak et al., 2018; Sencio et al., 2020). Alterations in gut microbiota have already been found not only in animal studies but also in clinical research (Gu et al., 2020; Zuo et al., 2020; Seibert et al., 2021; Yeoh et al., 2021; Blackett et al., 2022; Cuicchi et al., 2022). A limitation of this study is that the regulatory mechanism of intestinal infection during COVID-19 disease progression was not further investigated. Nevertheless, the established mouse model in this study showed similarities to COVID-19 patients in the tissue distribution of hACE2 expression and SARS-CoV-2 infection characteristics in both the lungs and intestines. Therefore, this model could be a useful tool for studying SARS-CoV-2 intestinal infection and developing preventive and therapeutic drugs for COVID-19 using different strategies.

## Data availability statement

The original contributions presented in the study are included in the article/supplementary material, further inquiries can be directed to the corresponding authors.

## Ethics statement

All experimental procedures involving mice in this study were approved by the Laboratory Animal Welfare and Ethics Committee of the Third Military Medical University (AMUWE20201373).

## Author contributions

JL conceived and planned the overall structure of the article. XZ, WS, YZ, and HG contributed to draft the manuscript and development of methodology. RW, PX, YKZ, MQ, and XD performed the experiments and prepared the figures and tables. JL, HW, and YG edited the manuscript. All authors contributed to the article and approved the submitted version.

## Funding

The project was supported by National Natural Science Foundation of China (Grant No. 81570497) and State Key Laboratory of Pathogen and Biosecurity (Academy of Military Medical Science, SKLPBS2106).

## Conflict of interest

The authors declare that the research was conducted in the absence of any commercial or financial relationships that could be construed as a potential conflict of interest.

## Publisher’s note

All claims expressed in this article are solely those of the authors and do not necessarily represent those of their affiliated organizations, or those of the publisher, the editors and the reviewers. Any product that may be evaluated in this article, or claim that may be made by its manufacturer, is not guaranteed or endorsed by the publisher.

## Abbreviations

COVID-19: Coronavirus disease 2019
SARS-CoV-2: severe acute respiratory syndrome coronavirus 2
hACE2: human angiotensin-converting enzyme 2
KI: knock-in
K18: epithelial cell cytokeratin-18
CRISPR: clustered regularly interspaced palindromic repeats
sgRNAs: single guide RNAs
WT: wild-type
MT: mutation
PFU: plaque-forming units
dpi: days post-infection
qPCR: quantitative PCR
E: envelope gene
S: spike protein
H&E: hematoxylin and eosin
CDS: coding sequence
